# Abnormal Resting-State Functional Connectivity Strength in Mild Cognitive Impairment and Its Conversion to Alzheimer's Disease

**DOI:** 10.1155/2016/4680972

**Published:** 2015-12-30

**Authors:** Yuxia Li, Xiaoni Wang, Yongqiu Li, Yu Sun, Can Sheng, Hongyan Li, Xuanyu Li, Yang Yu, Guanqun Chen, Xiaochen Hu, Bin Jing, Defeng Wang, Kuncheng Li, Frank Jessen, Mingrui Xia, Ying Han

**Affiliations:** ^1^Department of Neurology, XuanWu Hospital of Capital Medical University, Beijing 100053, China; ^2^Department of Neurology, Tangshan Gongren Hospital, Tangshan 063000, China; ^3^Department of Psychiatry and Psychotherapy, University Hospital of Cologne, Kerpener Strasse 62, 50937 Cologne, Germany; ^4^School of Biomedical Engineering, Capital Medical University, Beijing 100069, China; ^5^Research Center for Medical Image Computing, Department of Imaging and Interventional Radiology, The Chinese University of Hong Kong, Shatin, Newterritories 000852, Hong Kong; ^6^Department of Radiology, XuanWu Hospital of Capital Medical University, Beijing 100053, China; ^7^State Key Laboratory of Cognitive Neuroscience and Learning and IDG/McGovern Institute for Brain Research, Beijing Normal University, Beijing 100875, China; ^8^Center of Alzheimer's Disease, Beijing Institute for Brain Disorders, Beijing 100053, China

## Abstract

Individuals diagnosed with mild cognitive impairment (MCI) are at high risk of transition to Alzheimer's disease (AD). However, little is known about functional characteristics of the conversion from MCI to AD. Resting-state functional magnetic resonance imaging was performed in 25 AD patients, 31 MCI patients, and 42 well-matched normal controls at baseline. Twenty-one of the 31 MCI patients converted to AD at approximately 24 months of follow-up. Functional connectivity strength (FCS) and seed-based functional connectivity analyses were used to assess the functional differences among the groups. Compared to controls, subjects with MCI and AD showed decreased FCS in the default-mode network and the occipital cortex. Importantly, the FCS of the left angular gyrus and middle occipital gyrus was significantly lower in MCI-converters as compared with MCI-nonconverters. Significantly decreased functional connectivity was found in MCI-converters compared to nonconverters between the left angular gyrus and bilateral inferior parietal lobules, dorsolateral prefrontal and lateral temporal cortices, and the left middle occipital gyrus and right middle occipital gyri. We demonstrated gradual but progressive functional changes during a median 2-year interval in patients converting from MCI to AD, which might serve as early indicators for the dysfunction and progression in the early stage of AD.

## 1. Introduction

Alzheimer's disease (AD), an irreversible neurodegenerative disease characterized by memory dysfunction, executive function decline, and multiple cognitive domain impairments, is one of the most financially costly diseases [1]. Since there is currently no effective treatment to stop or reverse the progression of AD, the research spotlight has turned to its predementia stage, specifically termed amnestic mild cognitive impairment (aMCI). For individuals with MCI due to AD (called “aMCI” or MCI in this paper for short), the development of AD is a high risk factor that the rate of MCI conversion to AD reaches 10% to 15% annually [2]. Considering the urgent requirement for the identification of those MCI patients who are most likely to undergo rapid progression and conversion to AD, it is of great significance to investigate and discover the potential biomarkers for the early identification of the dysfunction and progression in the early stage of AD.

Magnetic resonance imaging, a noninvasive, nonradiation means for the mapping of both structures and functions of the human brain, is a promising avenue to investigate the progressive brain changes from MCI to AD [3, 4]. Structurally, studies have consistently found that the gray matter atrophy originally starts at the medial temporal lobe, spreads along the midline of the cerebral cortex, and finally extents to the whole brain during the progress from MCI to AD [5–7]. Functionally, however, investigations have yielded limited functional biomarkers that predict the progression from MCI to AD, except the consistent identification of the changes of resting-state functional connectivity (RSFC) of the default-mode network (DMN) in AD [8, 9]. However, deficits in RSFC are not confined to the DMN in patients with MCI converting to AD [10]. Furthermore, it is not clear whether other brain regions participate in the conversion to AD.

Most previous studies have focused on the AD- or MCI-related functional connectivity changes of specific predefined regions of interest, such as posterior cingulate cortex and thalamus [11, 12]. Given the complex pathology and widespread functional abnormalities in AD and MCI, it would be of great interest to examine differences between MCI-converters (MCI-c) and MCI-nonconverters (MCI-nc) within a whole-brain range. Here, we used resting-state functional magnetic resonance imaging (R-fMRI) data and functional connectivity strength (FCS), computed as the sum of connections between a given voxel and all other voxels [13–15], to detect the functional differences among AD, MCI, and normal controls and especially between MCI patients who converted to AD (MCI-c) and MCI-nc. We sought to determine (1) whether there exists an AD-related progressive abnormality pattern on the whole-brain functional connectivity strength in MCI patients and (2) if so whether these changes are different between MCI-c and MCI-nc groups and are related to their clinical behaviors.

## 2. Materials and Methods

### 2.1. Participants

The study was approved by the Research Ethics Review Board of XuanWu Hospital (ClinicalTrials.gov Identifier: NCT02353845). A total of 98 right-handed subjects were recruited in the study including 25 AD patients, 31 MCI patients, and 42 well-matched cognitive normal controls. All AD and MCI patients were recruited at the memory clinic of the Neurology Department, XuanWu Hospital, Capital Medical University, Beijing, China. Control subjects were recruited from the local community via broadcast and advertisements. Diagnoses of MCI due to AD were made by experienced neurologists using Petersen's criteria [16]. The diagnosis of AD fulfilled the published diagnostic criteria [17]. Controls were screened as described in the* Structured Interview for DSM-IV Nonpatient Edition* [18] to confirm the life-long absence of psychiatric and neurological illness. Inclusion criteria for MCI due to AD included the following: (1) memory complaint, preferably confirmed by an informant; (2) objective memory impairment, (cutoff points of Mini-Mental State Examination (MMSE) score [19]: 19 (no formal education), 22 (1 to 6 years of education), and 26 (7 or more years of education); cutoff points of Montreal Cognitive Assessment (MoCA) [20]: 13 (no formal education), 19 (1 to 6 years of education), and 24 (7 or more years of education); cutoff point of auditory verbal learning test- (AVLT-) delayed recall [21]: 6); (3) no or minimal impairment of daily life activities; (4) a Clinical Dementia Rating (CDR) [22] score of 0.5; (5) being free from dementia according to the* Diagnostic and Statistical Manual of Mental Disorders, Fourth Edition*, revised (DSM-IV-R) [18]; (6) hippocampal atrophy confirmed by structural MRI; and (7) the Han nationality, right-handed (the Edinburgh handedness scale score [23] >40 points). The exclusion criteria applied to all subjects with contraindications for MRI; also excluded were those with histories of stroke, psychiatric disease, neurological disorder, alcohol or drug abuse, and systemic disease such as severe anemia, thyroid dysfunction, syphilis, or Acquired Immune Deficiency Syndrome. All subjects underwent a standardized clinical and neuropsychological evaluation, including the MMSE, MoCA, clock drawing test (CDT), AVLT, activities of daily living scale, Hachinski Ischemic Scaling, Hamilton Depression Scale, and CDR. Second, the quality of the whole-brain resting-state functional MRI images was inspected by an experienced neuroradiologist. Third, after a mean follow-up period of 24 months (ranging from 11 months to 48 months), subjects again underwent the entire clinical and neuropsychological assessment. All subjects underwent a follow-up review of approximately 24 months, and according to the diagnosis in the follow-up stage, MCI subjects were divided into converters to AD (MCI-c, *n* = 21) and nonconverters (MCI-nc, *n* = 10).

### 2.2. Image Acquisition

All participants were scanned within a single session on a 3.0T Trio Siemens scanner at XuanWu Hospital, Capital Medical University. Resting-state functional images were collected using an echo-planar imaging sequence with the following parameters: repetition time (TR) = 2000 ms, echo time (TE) = 40 ms, flip angle (FA) = 90°, number of slices = 28, slice thickness = 4 mm, gap = 1 mm, voxel size = 4 × 4 × 4 mm^3^, and matrix = 64 × 64. Participants were asked to lie quietly in the scanner with their eyes closed during data acquisition. Each scan lasted for 478 s. For registration purposes, high-resolution anatomical images were acquired using a 3D magnetization-prepared rapid gradient echo (MPRAGE) T1-weighted sequence with the following parameters: TR = 1900 ms, TE = 2.2 ms, inversion time (TI) = 900 ms, FA = 9°, number of slices = 176, slice thickness = 1 mm, voxel size = 1 × 1 × 1 mm^3^, and matrix = 256 × 256.

### 2.3. Data Analysis

#### 2.3.1. Image Preprocessing

Image preprocessing was performed by using SPM8 (http://www.fil.ion.ucl.ac.uk/spm/) and Data Processing Assistant for R-fMRI [24]. The preprocessing procedures were performed including removal of the first 10 volumes, slice timing, and head motion correction. All data used in this study satisfied the criteria of spatial movement in any direction < 3 mm or 3° and the subjects demonstrated no significant group differences in the head motion parameters (i.e., three translation and three rotation parameters). To normalize the fMRI data spatially, the T1-weighted images were firstly registered to the mean functional data, and the resulting aligned T1 data set was segmented and transformed into MNI space using the DARTEL toolbox [25] and a group template was generated. Next, the motion-corrected functional volumes were specially normalized to the group template using the transfer parameter estimated by DARTEL segmentation and resampled to 3 mm isotropic voxels. Further, the functional images were spatially smoothed with a 4 mm Gaussian kernel. The linear detrend and temporal band-pass filtering (0.01–0.08 Hz) was performed to reduce the influences of low-frequency drift and high-frequency physiological noise. Finally, several nuisance signals were regressed out from the data, including the six motion parameters, the global, the white matter, and the cerebrospinal fluid signals.

#### 2.3.2. Whole-Brain Functional Connectivity Strength

To perform the whole-brain RSFC analysis, Pearson's correlations between the time courses of any pairs of voxels were first computed, resulting in a whole-brain connectivity matrix for each participant. This procedure was limited within a gray matter (GM) mask, which was generated by thresholding (cutoff = 0.2) the mean map of all GM maps involving all subjects without cerebellum. These individual correlation matrices were then transformed as a *z*-score matrix by using Fisher's *r*-to-*z* transformation to improve normality. We computed the FCS as the sum of the connections between a given voxel and all other GM voxels. This computation was conservatively restricted to connections with a correlation coefficient above 0.2, which could eliminate the weak correlations possibly arising from noise.

#### 2.3.3. Seed-Based Functional Connectivity

To examine the detailed RSFC differences between MCI-c and MCI-nc, we performed seed-based connectivity analyses, using the clusters showing significant between-group difference on FCS as the seeds (i.e., left angular gyrus and middle occipital gyrus). Briefly, the mean time course within each seed was extracted by averaging the time courses of all the voxels belonging to the seed. Subsequently, the mean time course was further used to compute correlation coefficients with the time courses of all GM voxels. Notably, the computation was constrained within a custom GM mask that was made by thresholding (a probability threshold of 0.2) the GM probability map obtained in DARTEL segmentation. The resulting correlation coefficients were then converted to *z*-scores using Fisher's *r*-to-*z* transform to improve normality. For each MCI patient, we obtained two *z*-score maps indicative of the intrinsic RSFC patterns of the two seeds (i.e., left angular gyrus and middle occipital gyrus) based on the previous results of the group difference on FCS. Notably, given the ambiguous biological interpretations of negative functional connections, the statistical analysis for RSFC was restricted to positive connections.

#### 2.3.4. Statistical Analysis

A one-way analysis of covariance (ANCOVA) was performed to determine the main effect of groups on FCS, with age and gender as covariates, followed by two-sample *t*-tests* post hoc* analyses. The result for ANCOVA was thresholded at *P* < 0.05 with a cluster size of 1350 mm^3^, corresponding to a corrected *P* < 0.05. The two-sample *t*-tests* post hoc* analyses were performed within the regions showing significant group effects, and the threshold was set at *P* < 0.05 with a cluster size of 324 mm^3^, corresponding to a corrected *P* < 0.05. Furthermore, in the AD pathology-related group, to determine the difference between MCI-c and MCI-nc, we performed a two-sample *t*-test on FCS maps of the MCI-c and MCI-nc within the regions showing significant differences of AD against controls. The significant level was set at *P* < 0.05 with cluster size of 216 mm^3^, corresponding to a corrected *P* < 0.05. All the cluster sizes were determined by Monte Carlo simulations [26] using the REST AlphaSim utility [27].

The two-sample *t*-tests were performed on the RSFC maps for each seed, with age and gender as covariates. The significant level was set at *P* < 0.05 with a cluster size of 1350 mm^3^, corresponding to a corrected *P* < 0.05. The analysis mask was generated by selecting the voxels that showed significant positive RSFC in any of the two groups. To investigate the relationship between FCS and cognitive behavior, we performed general linear model analysis (dependent variable: FCS; independent variable: clinical variables, including MMSE, MoCA, AVLT-immediate recall, AVLT-delayed recall, and AVLT-delayed recognition) in the combined AD and MCI group with age and gender treated as covariates within the regions showing group effect. The statistical threshold was set to *P* < 0.05 with a cluster size of 324 mm^3^, which corresponded to a corrected *P* < 0.05.

#### 2.3.5. Discriminate Analysis

To assess whether the discovered differences of FCS and RSFC between MCI-c and MCI-nc could serve as the features to identify MCI-c patients from MCI-nc patients, we used support vector machine (SVM) as classifier to distinguish patients of the two groups. The features were selected as the values of voxels showing significant between-group differences, including the FCS and the whole-brain functional connectivity of the left angular gyrus and middle occipital gyrus. The leave-one-out cross-validation (LOOCV) was then used to estimate the performance of our classifier. In LOOCV, each sample was designated as the test sample, while the remaining samples were used to train the classifier. Accuracy, sensitivity, and specificity can be defined on the basis of prediction results of LOOCV to quantify the performance of the classifier:

(1) accuracy=TP+TNTP+FN+TN+FP,sensitivity=TPTP+FN,specificity=TNTN+FP,

where TP, FN, TN, and FP denoted the number of MCI-c patients correctly predicted, the number of MCI-c patients classified as MCI-nc patients, the number of MCI-nc patients correctly predicted, and the number of MCI-nc patients classified as MCI-c patients, respectively.

#### 2.3.6. Validations

Given that the results of the FCS analysis might be influenced by several methodological choices (e.g., correlation threshold, head motion, and removal of global signal), we conducted the following procedures and recompared the FCS within left angular gyrus and middle occipital gyrus between the two MCI groups. (i) Change of correlation thresholds. In the initial analysis, a correlation coefficient threshold of 0.2 was used during the FCS analysis. To determine whether the FCS results depend on the selection of correlation thresholds, the other two different correlation thresholds (i.e., 0.1 and 0.3) were used to recompute the FCS maps. There resultant FCS maps were then used to perform the statistical analyses, respectively. (ii) Include the head motion parameter into statistical analysis. The influences of head motion on RSFC have been reported by several studies recently [28–30]. Although we observed no significant differences between any pairs of the groups in the maximum movements at each direction, we cautiously evaluated the head motion effects on our results by calculating the frame-wise displacement (FD) of our data [30] and further compared the group difference by adding FD as an additional nuisance covariate. (iii) Do not use global signal regression (GSR). Whether the global mean signal should be removed is currently still debatable in the preprocessing procedure of the R-fMRI images. Some studies suggested that the global signal should be removed [31], as it was confounded with physiological noise [32], whereas several other studies [33, 34] indicated that the GSR could introduce negative correlations and therefore alter the intrinsic architecture of the brain network. To examine whether the process of GSR changes our results, the data was reanalyzed without using GSR in the preprocessing steps.

## 3. Results

### 3.1. Demographics and Neuropsychological Tests

Clinical and demographic data for the 98 participants are presented in Tables 1 and 2. Some of these data were used previously to detect functional brain abnormalities in MCI patients [35, 36]. There were no significant differences among MCI, AD, or controls with respect to age (*P* = 0.22), gender (*P* = 0.67), and years of education (*P* = 0.10). However, the clinical variables, including the MMSE, MoCA, CDT, AVLT-immediate recall (AVLT-I), AVLT-delayed recall (AVLT-D), and AVLT-delayed recognition (AVLT-R), differed significantly among the three groups (*P* < 0.0001 for all comparisons), with an ascending order of AD, MCI, and controls. Furthermore, there were no significant differences between MCI-c and MCI-nc groups in age, gender, years of education, or any of the clinical or neuropsychological variables (*P* > 0.12 for all comparisons). However, after a mean follow-up period of 24 months, the MMSE and AVLT-I were significantly lower in the MCI-c group than in the MCI-nc group (both *P*  values < 0.02), and the AVLT-D, AVLT-R, and MoCA were marginally lower in the MCI-c group (all *P*  values < 0.1).

### 3.2. Whole-Brain Functional Connectivity Strength

**Figure 1:**
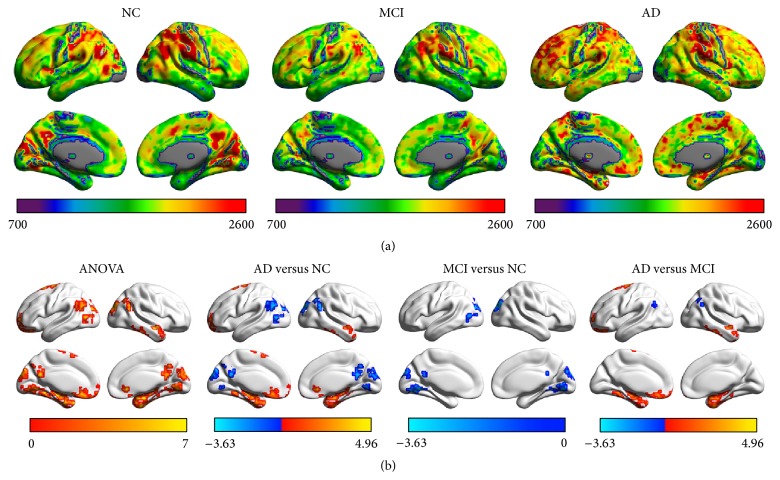
The FCS in AD, MCI, and control groups. (a) The images show the mean FCS in AD, MCI, and control groups. The color bar at the bottom of each picture represents the FCS value for each group. (b) The images demonstrated the significant differences among the three groups and within each pair of the groups at baseline. The color bar at the bottom of each picture represents either *F* values for ANOVA or *T* values for* post hoc t*-tests.

The spatial patterns of FCS were remarkably similar across the MCI, AD, and control groups by visual inspection, in spite of different strengths. Regions with high FCS were mostly located in the DMN (mainly involving the medial prefrontal cortex, precuneus, posterior cingulate cortex, and inferior parietal lobule), anterior insula, sensorimotor, and visual cortices (Figure 1(a)). The FCS patterns were similar to those observed in previous studies [13–15].

**Figure 2:**
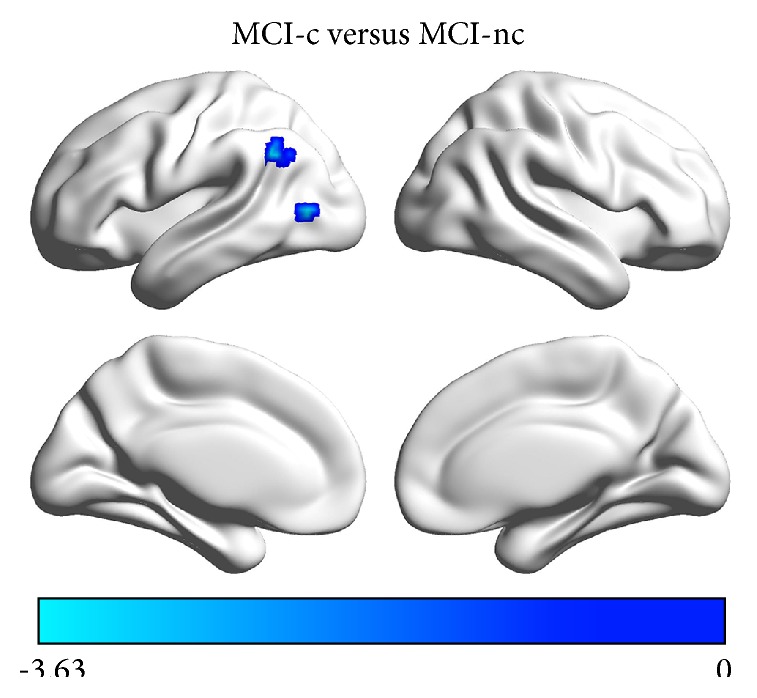
The FCS differences between MCI-c and MCI-nc groups. The FCS of the left angular gyrus and middle occipital gyrus were significantly lower in the MCI-c group compared with the MCI-nc group. The color bar represents the *T* values for the two-sample *t*-test between MCI-c and MCI-nc groups.

Significant group differences of FCS among the MCI, AD, and control groups were observed in bilateral precuneus/posterior cingulate cortices (PCu/PCC), bilateral parahippocampal cortices, bilateral angular gyri, right temporal pole, left superior frontal gyrus, left orbitofrontal cortex, bilateral lingual gyri, and left middle occipital gyrus (Figure 1(b), Table 3). The* post hoc* analysis revealed that (i) the MCI showed decreased FCS in bilateral PCu/PCC, bilateral lingual gyri, and left middle occipital gyrus, as compared to controls (Figure 1(b)); (ii) the AD group exhibited lower FCS than controls in bilateral PCu/PCC, bilateral angular gyri, bilateral lingual gyri, and left middle occipital gyrus, but greater FCS in bilateral parahippocampal cortices, right temporal pole, left superior frontal gyrus, and left orbitofrontal cortex (Figure 1(b)); and (iii) the AD group had significantly lower FCS in bilateral angular gyri but higher FCS in bilateral parahippocampal cortices, right temporal pole, left superior frontal gyrus, and left orbitofrontal cortex than the MCI group (Figure 1(b)). In the comparison between MCI-c and MCI-nc, the FCS of the left angular gyrus and middle occipital gyrus were significantly lower in the MCI-c group (Figure 2).

### 3.3. Seed-Based Functional Connectivity

**Figure 3:**
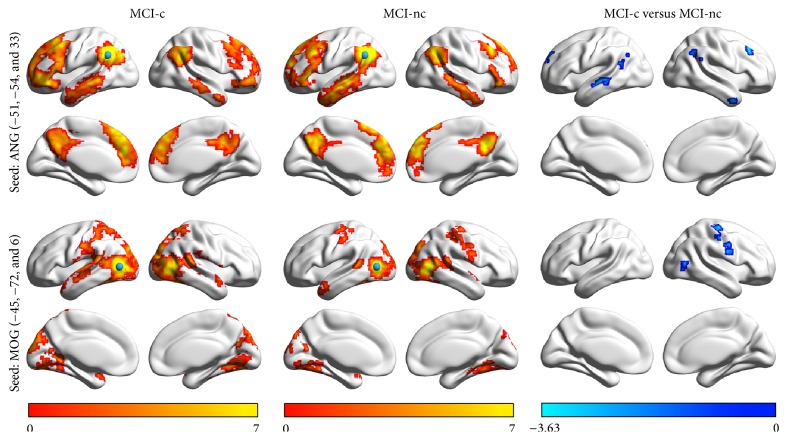
The seed-based RSFC analysis between MCI-c and MCI-nc. The left and middle column present the within group RSFC pattern for the left angular gyrus and the left middle occipital gyrus. Between-group comparison on the right column revealed that comparing to MCI-nc group the MCI-c group had significantly decreased functional connectivity between the left angular gyrus and bilateral dlPFC and lateral temporal cortices and between the left middle occipital gyrus and right central sulci and right middle occipital gyrus. The color bars at the bottom represent the *T* value for either the one-sample *t*-test or two-sample *t*-test.

To examine the detailed difference in RSFC of the left angular gyrus and middle occipital gyrus, we generated whole-brain RSFC maps of each region in the two MCI groups. The spatial patterns of the whole-brain RSFC for each seed region were similar across the two groups. The left angular gyrus was functionally connected with the default-mode regions, including the PCu/PCC, medial prefrontal cortex, inferior parietal lobule (IPL), dorsolateral prefrontal cortex (dlPFC), and lateral temporal cortex, whereas the left middle occipital gyrus exhibited RSFC with the sensorimotor and visual cortices. Between-group comparisons revealed that the MCI-c patients had significantly decreased RSFC between the left angular gyrus and bilateral IPL, dlPFC and lateral temporal cortices, and the left middle occipital gyrus and right central sulci and right middle occipital gyrus, as compared to the MCI-nc group (Figure 3).

### 3.4. Discriminate Analysis

The SVM method achieved a classification accuracy of 80.6%, with sensitivity of 70.0% and specificity of 85.7% in distinguishing MCI-c patients from MCI-nc patients, suggesting the potential capacity of the functional metrics left angular gyrus and middle occipital gyrus in predicting converting from MCI to AD.

### 3.5. Correlations between Functional Connectivity Strength and Neuropsychological Variables

**Figure 4:**
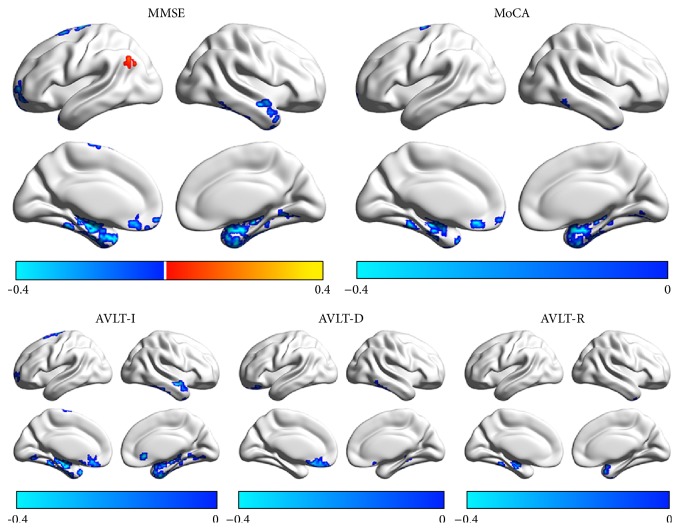
The correlation between FCS and neuropsychological scores in patients. The color bar represents the *r* value. AVLT-I, auditory verbal learning test-immediate recall; AVLT-D, auditory verbal learning test-delayed recall; AVLT-R, auditory verbal learning test-recognition; MMSE, Mini-Mental State Examination; MoCA, Montreal Cognitive Assessment.

Significant positive correlation was found between FCS of the left angular gyrus and MMSE. The FCS of the medial temporal cortices was significantly negatively correlated with all the clinical and neuropsychological variables. Additionally, significant negative correlations were observed between the FCS of left dorsal frontal and right lateral temporal cortices and MMSE, MoCA and AVLT-I, and the FCS of ventral genu anterior cingulate cortex and MMSE, MoCA, AVLT-I, and AVLT-D (Figure 4).

### 3.6. Validations

We found that the differences on FCS in the left angular gyrus and middle occipital gyrus between two MCI subgroups were quite stable across different process procedures. The FCS in these two regions remained significantly lower value in MCI-c group under different network correlation thresholds (for threshold 0.1: angular gyrus, *t* = −3.73, *P* = 0.00094; middle occipital gyrus, *t* = −3.12, *P* = 0.0044; for threshold 0.3: *t* = −3.81, *P* = 0.00077; middle occipital gyrus, *t* = 3.14, *P* = 0.0042). There are no significant group differences in the movement parameter FD (*P* = 0.6). FCS differences were unchanged after adding FD as an additional covariate to the reanalysis (angular gyrus, *t* = 3.81, *P* = 0.00085; middle occipital gyrus, *t* = 3.05, *P* = 0.0053). However, these group differences could not be identified in case the global signal was retained (*P* > 0.88 for all comparisons) in the preprocessing, suggesting that the pathological differences might be buried into systematic and physiological noise.

## 4. Discussion

The present longitudinal study was designed to use R-fMRI for the identification of valuable imaging markers in patients with MCI and AD for predicting conversion from MCI to AD dementia in a mean follow-up of two years. We demonstrated that (i) the MCI group showed decreased FCS in the default-mode regions and occipital cortex, as compared to normal controls at baseline; however, the AD group exhibited simultaneously lower and higher FCS than the MCI and NC group; (ii) the FCS of the left angular gyrus and middle occipital gyrus was significantly lower in the MCI-c group than MCI-nc group. Finally, FCS of several brain regions correlated with clinical and neuropsychological scores.

### 4.1. Features of Functional Connectivity Strength in Subjects with AD and MCI

AD is considered as a disconnection syndrome, and as previous studies showed, AD patients have abnormal RSFC between several brain regions, especially within the DMN [37, 38]. Here, the use of FCS method confirmed progressive functional changes in the DMN from MCI to AD. Our study extended previous finding into MCI patients and provided additional evidence of the progressive features of brain function in subjects with MCI who develop AD.

We observed that patients with MCI and AD showed decreased FCS in the DMN (including bilateral PCu/PCC) and occipital cortex as compared to controls, in line with previous studies [9, 37–41]. At the early stage of AD, the declines of episodic memory of the patients have been associated with the structural and functional defects in the DMN, which might be due to their underlying accumulations of beta-amyloid plaques [42] and decreased metabolic activity [43]. Studies reported that the decline of RSFC was related to the low neuropsychological assessment scores and to higher conversion rates from MCI to AD [9] or to AD progression [44]. Furthermore, the current study also validated the results of our previous research denoting impairment in the DMN of patients with AD and MCI [35, 36, 45]. Beyond these findings, the occipital cortex (including bilateral lingual gyri and left middle occipital gyrus) manifested decreases of FCS in patients with AD and even MCI. The lingual gyrus and middle occipital gyrus are located in the visual network and are associated with the processing of visual memory and visuospatial function [46, 47]. Our results provided the potential brain bases for the impaired multiple cognitive domains in MCI and AD, which is in consistency with previous study showing the decline of functional connectivity of the occipital cortex in AD and MCI patients [48].

Other than the brain regions cited above, the bilateral angular gyri manifested reductions in FCS in bearers of AD rather than those with MCI, which is consistent with several previous studies [49, 50]. The angular gyrus is an important brain region of the inferior parietal lobule in the DMN, and its impairments are highly correlated with damage of multiple cognitive domains to identify direction and presence of alexia, agraphia, and dyscalculia in AD patients [51]. A recent study showed decreased RSFC between the left angular gyrus and right thalamus and such an alteration of the thalamo-DMN circuit was also linked to the disease severity in AD and MCI patients [49]. Specifically, in the current study, FCS of the left angular gyrus was positively related to MMSE of patients, suggesting that FCS of the left angular gyri may be a potential imaging marker for monitoring disease progression.

Comparing with MCI patients, AD exhibited increased FCS mainly involving the frontal lobe (including left superior frontal gyrus and orbitofrontal cortex) and temporal lobe (including bilateral parahippocampal cortices and right temporal pole). A notable finding in this study was significant negative correlations between the FCS of left dlPFC, ventral genu anterior cingulate cortex, right lateral temporal cortices, and neuropsychological scores such as MMSE, MoCA, and AVLT-I. A possible mechanistic explanation for these increased FCS could be the compensation mechanism [48, 52, 53] that acts to counterbalance regional deficits in function [54]. Compensatory mechanisms accompany the impairments seen during the interval when a patient with MCI progresses to AD [11]. A study utilizing single photon emission computed tomography demonstrated the compensation mechanism in the medial temporal lobe in AD with the phenomenon of high perfusion in the neocortex along with hypoperfusion [55]. Besides, synaptic loss was related to the cognitive decline in AD and compensation mechanisms via maintaining the activity levels of neural circuits that could otherwise reduce the impairment of cognitive function induced by synaptic loss [56]. Here, the observation of the increases of FCS in AD rather than in MCI patients suggests that AD patients could utilize additional brain connectivity for cognitive functions, presumably as a compensatory mechanism for cognitive decline.

### 4.2. The Diversity of Functional Connectivity between MCI-c and MCI-nc

Compared to the MCI-nc group, the MCI-c patients had significantly decreased RSFC between the left angular gyrus and bilateral IPL, dlPFC and lateral temporal cortices, and the left middle occipital gyrus and right central sulci and right middle occipital gyrus. IPL (including the angular gyrus and supramarginal gyrus) and dlPFC were the key component in the DMN, and many previous studies have suggested that the lesion in IPL was closely related to AD, especially in MCI [49, 57, 58]. Interestingly, this result explained the reductions of FCS in the bilateral angular gyri in AD rather than in MCI in the current study, which suggested that the decline of FCS in the angular gyrus in MCI-c group may predict a more serious disease closer to AD dementia. Furthermore, several previous studies found decreased RSFC in the dlPFC in early AD, which involves in a series of cognitive functions, including working memory, decision making, and executive controls [38, 59].

Studies have consistently identified structural and metabolic abnormality in the lateral temporal cortex during stage of AD and MCI [60–62]: Li and colleagues found significant reductions in gray matter volume of the left lateral superior temporal gyrus in patients with MCI [62], suggesting that the lateral temporal lobe was impaired in the early stage of MCI. Additionally, we revealed the decline of RSFC of the occipital cortex (middle occipital gyrus) in MCI-c group, compared to MCI-nc. It should be noted that the abnormality in the occipital cortex has gained less attention than the DMN regions in previous studies in MCI developing to AD [8, 9]. Activity and functional connectivity of the occipital lobe are highly associated with visual hallucination [63], visual memory [46], and visuospatial function [47]. Therefore, the functional impairments of occipital cortex could suggest the probable multiple cognitive domains damage, especially the visual cognitive declines in patients converting to AD. Interestingly, a recent study on atrophy patterns of various phenotypes of AD showed the atrophy in the visual network to be dominant in the AD phenotype with posterior cortical atrophy [64]. A more refined differential diagnosis of various AD phenotypes in the future is necessary to delineate the probable relationship between decreased FCS in occipital cortex and specific functional impairment in AD. In summary, the functional alterations of DMN and visual cortex demonstrated imaging impairments in the conversion from MCI to AD and might provide potential biomarkers for predicting MCI progression.

### 4.3. Further Considerations

Several limitations of the present study require further considerations. First, the clinical criteria for the recruitment of MCI did not classify different subtypes of aMCI patients (single or multiple domain impairment), which introduces the clinical heterogeneity of our data set. Future studies could focus on the differences and conversation of different types of MCI to better characterize the pathology of aMCI. Second, in the present longitudinal study, the conversion rate from MCI to AD was relatively high (33.87% annually), which might be because these patients came to the clinic with obvious memory symptoms at a late stage of MCI. Future studies aiming at the longitudinal database with larger sample size including early MCI and even preclinical stage of AD with multimodal imaging and biophysical data (e.g., Alzheimer's Disease Neuroimaging Initiative (ADNI) database) are of great importance to investigate the progression of AD. Third, the MCI-nonconverters in the current study were just stable in the follow-up stage (about two years) and whether they would convert to AD in the future remains unknown. Therefore, we planned to continue to track these patients to observe the dynamic changes and delineate the progressing trajectory on brain changes during the AD progression. Fourth, we noticed that the FCS differences between MCI-c and MCI-nc could not be identified in case the global signal was retained in the preprocessing. Recent studies have demonstrated raised variability of global signal in schizophrenia but not bipolar illness, suggesting the potential specific association between brain disorders and global signal [65]. Future studies focusing on the global signal in AD could further reveal the deep relationship between the pathology of AD and physiological signals. Finally, the current study was concentrated on the R-fMRI functional connectivity of the whole brain. Further studies that simultaneously combine the R-fMRI, diffusion tensor MRI, and other biophysical data would reveal structural and biological substrates underlying these functional deficits in AD and MCI.

## 5. Conclusions

In conclusion, we demonstrated gradual but progressive functional changes during median 2-year interval in patients converting from MCI to AD, which might serve as early indicators for the dysfunction and progression in the early stage of AD.

## Figures and Tables

**Table 1:** Demographics and clinical characteristics of the participants.

	AD (*n* = 25)	MCI (*n* = 31)	Control (*n* = 42)	*F* or χ^2^ value	*P* value
Age (years)	51–88 (69.4 ± 11.1)	50–82 (67.9 ± 9.5)	51–79 (65.6 ± 7.1)	*F*_(2,95)_ = 1.52	0.22^a^
Gender (M/F)	9/16	14/17	15/27	χ_(2)_^2^ = 1.52	0.67^b^
Education years	0–17 (8.3 ± 5.4)	0–21 (10.1 ± 5)	0–18 (11.1 ± 4.9)	*F*_(2,95)_ = 2.4	0.10^a^
MMSE	6–24 (16.8 ± 4.7)	17–29 (23.5 ± 2.9)	20–30 (28.0 ± 2.3)	*F*_(2,95)_ = 93.04	<0.0001^a^
MoCA^c^	5–22 (12.8 ± 4.8)	9–24 (18.3 ± 3.9)	19–30 (26.0 ± 2.8)	*F*_(2,73)_ = 81.32	<0.0001^a^
CDT^d^	0–3 (1.7 ± 1.1)	0–3 (1.8 ± 0.8)	1–3 (2.9 ± 0.4)	*F*_(2,87)_ = 23.39	<0.0001^a^
AVLT-I	0–5.7 (3.6 ± 1.5)	2–7 (4.6 ± 1.3)	6–14.7 (9.3 ± 2.1)	*F*_(2,95)_ = 108.87	<0.0001^a^
AVLT-D	0–4 (0.6 ± 1.1)	0–7 (2.7 ± 2.2)	4–15 (10.4 ± 3.0)	*F*_(2,95)_ = 159.79	<0.0001^a^
AVLT-R	−2–8 (3.4 ± 3.1)	−3–13 (7.1 ± 3.9)	7–15 (12.4 ± 2.1)	*F*_(2,95)_ = 72.48	<0.0001^a^

Data are presented as the range of minimum–maximum (mean ± SD).

AD, Alzheimer's disease; MCI, mild cognitive impairment; MMSE, Mini-Mental State Examination; MoCA, Montreal Cognitive Assessment; CDT, clock drawing test; AVLT-I, auditory verbal learning test-immediate recall; AVLT-D, auditory verbal learning test-delayed recall; AVLT-R, auditory verbal learning test-recognition; MCI-c, mild cognitive impairment converter; MCI-nc, mild cognitive impairment nonconverter.

^a^The *P* value was obtained by one-way ANOVA.

^b^The *P* value was obtained by two-tailed Pearson chi-square test.

^c^MoCA included 24 AD patients, 22 MCI patients and 30 controls.

^d^CDT included 23 AD patients, 29 MCI patients and 38 controls.

**Table 2:** Demographics and clinical characteristics of MCI-c and MCI-nc patients.

	MCI-c (*n* = 21)	MCI-nc (*n* = 10)	*T* or χ^2^ value	*P* value
Baseline				
Age (years)	50–82 (68.6 ± 9.3)	50–78 (66.5 ± 10.4)	*T*_(29)_ = 0.57	0.57^b^
Gender (M/F)	11/10	3/7	*χ* ^2^_(1)_ = 4.19	0.24^a^
Education years	4–20 (10.5 ± 4.6)	0–21 (9.2 ± 5.9)	*T*_(29)_ = 0.66	0.52^b^
MMSE	17–28 (23.5 ± 2.9)	17–29 (23.6 ± 3.2)	*T*_(29)_ = −0.07	0.95^b^
MoCA^c^	9–24 (18.1 ± 3.7)	10–24 (18.7 ± 4.5)	*T*_(20)_ = −0.36	0.73^b^
CDT^d^	0–3 (1.7 ± 0.9)	1–3 (2.0 ± 0.7)	*T*_(27)_ = −0.79	0.44^b^
AVLT-I	2–6.7 (4.4 ± 1.2)	3–7 (4.9 ± 1.4)	*T*_(29)_ = −0.96	0.34^b^
AVLT-D	0–6 (2.3 ± 1.9)	0–7 (3.6 ± 2.6)	*T*_(29)_ = −1.60	0.12^b^
AVLT-R	−3–13 (6.6 ± 4.2)	1–12 (8.1 ± 3.1)	*T*_(29)_ = −0.99	0.33^b^
Follow-up^e^				
MMSE	9–28 (20.0 ± 4.2)	21–29 (24.4 ± 2.9)	*T*_(24)_ = −2.59	0.02^b^
MoCA	4–22 (15.8 ± 4.3)	12–24 (19.0 ± 4.0)	*T*_(24)_ = −1.69	0.10^b^
CDT	0–3 (1.7 ± 0.9)	1–3 (2.1 ± 0.7)	*T*_(24)_ = −1.23	0.23^b^
AVLT-I	0.7–6 (4.1 ± 1.3)	3.7–7.3 (5.5 ± 1.3)	*T*_(24)_ = −2.44	0.02^b^
AVLT-D	0–8 (1.7 ± 2.3)	0–6 (3.4 ± 2.0)	*T*_(24)_ = −1.99	0.06^b^
AVLT-R	0–13 (6.1 ± 3.8)	4–10 (8.3 ± 2.1)	*T*_(24)_ = −1.76	0.09^b^

Data are presented as the range of minimum–maximum (mean ± SD).

MCI, mild cognitive impairment; MMSE, Mini-Mental State Examination; MoCA, Montreal Cognitive Assessment; CDT, clock drawing test; AVLT-I, auditory verbal learning test-immediate recall; AVLT-D, auditory verbal learning test-delayed recall; AVLT-R, auditory verbal learning test-recognition; MCI-c, mild cognitive impairment converter; MCI-nc, mild cognitive impairment nonconverter.

^a^The *P* value was obtained by two-tailed Pearson chi-square test.

^b^The *P* value was obtained by two-sample two-tailed *t*-test.

^c^MoCA included 15 MCI-c and 7 MCI-nc patients.

^d^CDT included 19 MCI-c and 10 MCI-nc patients.

^e^The follow-up clinical scores included 17 MCI-c and 7 MCI-nc patients.

**Table 3:** Clusters showing significant group effects on FCS.

Number	Brain regions	Brodmann area	Cluster size (mm^3^)	Peak MNI coordinate			Max *F* score
				*x*	*y*	*z*	
1	R ITG/FG/HIP/PHG	20/37/28	12,582	51	−27	−27	11.19
2	L FG/HIP	20/38	9,153	−30	−42	−24	10.78
3	R TPOmid	38	2,376	54	−6	−15	8.62
4	L ORBsup	11	7,668	−3	12	−18	11.71
5	B LING	18	6,183	−18	−60	−6	8.75
6	L MOG	19	1,701	−45	−72	6	8.30
7	B PCC/PCu/CUN	31/7/19	12,339	−3	−51	21	9.52
8	R ANG	39	1,863	39	−63	42	13.09
9	L ANG	39	2,241	−54	−57	36	7.49
10	L SFG	6	2,349	−18	9	60	10.83

Significance level: *P* < 0.05; voxel size >1350 mm^3^; AlphaSim corrected *P* < 0.05.

B, bilateral; L, left; R, right; ITG, inferior temporal gyrus; FG, frontal gyrus; HIP, hippocampus; PHG, parahippocampal gyrus; TPOmid, middle temporopolar; ORB sup, superior orbitofrontal cortex; LING, lingual gyri; MOG, middle occipital gyrus; PCC, posterior cingulate cortices; PCu, precuneus; ANG, angular gyri; SFG, superior frontal gyrus; CUN, cuneus.
